# Lithium tetra­chlorido­aluminate, LiAlCl_4_: a new polymorph (*oP*12, *Pmn*2_1_) with Li^+^ in tetra­hedral inter­stices

**DOI:** 10.1107/S205698901701235X

**Published:** 2017-09-08

**Authors:** Stephan W. Prömper, Walter Frank

**Affiliations:** a Institut für Anorganische Chemie und Strukturchemie, Lehrstuhl II: Material- und Strukturforschung, Heinrich-Heine-Universität Düsseldorf, Universitätsstrasse 1, D-40225 Düsseldorf, Germany

**Keywords:** crystal structure, polymorphism, lithium tetra­chlorido­aluminate

## Abstract

Dissolving lithium chloride and aluminium chloride in boiling *para*- or *meta*-xylene and keeping the colourless solution at room temperature led to crystal growth of a new modification of lithium tetra­chlorido­aluminate, LiAlCl_4_, which represents a second modification (*oP*12, *Pmn*2_1_) of the ternary salt besides the long known monoclinic form [LiAlCl_4_(*mP*24, *P*2_1_/*c*); Mairesse *et al.* (1977 ▸). *Cryst. Struct. Commun.* **6**, 15–18]. The crystal structures of both modifications can be described as slightly distorted hexa­gonal closest packings of chloride anions. While the lithium cations in LiAlCl_4_(*mP*24) are in octa­hedral coordination and the aluminium and lithium ions in the solid of ortho­rhom­bic LiAlCl_4_ occupy tetra­hedral inter­stices with site symmetries *m* and 1, respectively, the lithium cation site being half-occupied (defect wurtz-stannite-type structure). From differential scanning calorimetry (DSC) measurements, no evidence for a phase transition of the ortho­rhom­bic modification is found until the material melts at 148 °C (*T*_peak_ = 152 °C). The melting point is nearly identical to the literature data for LiAlCl_4_(*mP*24) [146 °C; Weppner & Huggins (1976 ▸). *J. Electrochem. Soc.* **124**, 35–38]. From the melts of both polymorphs, the monoclinic modification recrystallizes.

## Abstract 2

The new polymorph of lithium tetra­chlorido­aluminate, LiAlCl_4_, adopts a defect wurtz-stannite-type of structure and is metastable.

## Chemical context   

The series of known crystal structures of alkali metal tetra­chlorido­aluminates *M*AlCl_4_, with *M* = Li (Mairesse *et al.*, 1977 ▸), Na (Baenziger, 1951 ▸), K (Mairesse *et al.*, 1978*a* ▸), Rb (Mairesse *et al.*, 1979 ▸) and Cs (Gearhart *et al.*, 1975 ▸; Mairesse *et al.*, 1979 ▸) was completed about 40 years ago and comparative structural studies were made (Mairesse *et al.*, 1979 ▸; Meyer & Schwan, 1980 ▸). With respect to ionic conductivity, both solid lithium tetra­chlorido­aluminate [LiAlCl_4_(*mP*24, *P*2_1_/*c*); Mairesse *et al.*, 1977 ▸] and melts of the salt were investigated (Weppner & Huggins, 1976 ▸, 1977 ▸). Besides the importance of common commercial lithium–thionyl chloride battery systems (Winter & Brodd, 2004 ▸), recently published studies on the conductivity of LiAlCl_4_ in dimethyl carbonate or mixtures with ethyl­ene carbonate (Scholz *et al.*, 2015 ▸) indicate that the substance is of continous inter­est. In the course of our on-going studies on arene complexation of main group metals (Frank, 1990 ▸; Frank *et al.*, 1987 ▸, 1996 ▸; Frank & Wittmer, 1997 ▸; Kugel, 2004 ▸; Bredenhagen, 2014 ▸), we isolated a new polymorph of LiAlCl_4_(*oP*12, *Pmn*2_1_) from mixtures of lithium chloride and aluminium chloride in boiling *para*- or *meta*-xylene, determined its crystal structure by single-crystal X-ray diffraction and unequivocally proved polymorphism of this ternary compound.

## Structural commentary   

**Figure 1:**
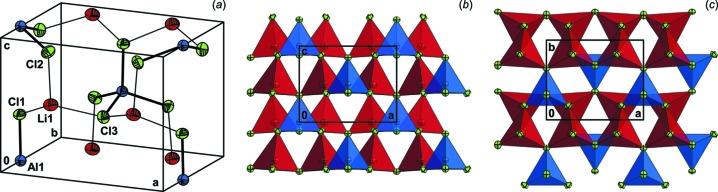
(*a*) The unit cell of the crystal structure of the title compound, with displacement ellipsoids drawn at the 50% probability level; (*b)* a view of the crystal structure in polyhedral representation perpendicular to a stacking direction ([010]) of the slightly distorted hexa­gonal closest packing of chloride anions; (*c)* a view of the crystal structure along [00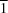].

LiAlCl_4_(*oP*12, *Pmn*2_1_) crystallizes in a defect wurtz-stannite-type structure, an ortho­rhom­bic superstructure of the wurtzite-type structure, known from quaternary compounds of the type Cu_2_ *M* ^II^ *M* ^IV^ *M*_4_^VI^ (*M* ^II^ = Mn, Fe, Co, Zn, Cd, Hg; *M* ^IV^ = Si, Ge, Sn; *M* ^VI^ = S, Se; except selenides of cobalt; Schäfer & Nitsche, 1977 ▸). The unit cell of the title compound contains four chloride anions and two aluminium cations, located in special positions (Wyckoff position 2*a*), as well as two lithium cations and another four chloride anions in general positions (4*b*), with the lithium site being half occupied, *i.e.* the asymmetric unit of the crystal structure is defined by half a tetra­chlorido­aluminate anion and one half-occupied lithium ion (Fig. 1 ▸ *a*).

The crystal structures of the title compound, as well as of the monoclinic modification of lithium tetra­chlorido­aluminate, can be described as slightly distorted hexa­gonal closest packings of chloride anions. While the lithium cations in LiAlCl_4_(*mP*24) are in octa­hedral coordination (Mairesse *et al.*, 1977 ▸), the aluminium and lithium ions in the solid of ortho­rhom­bic LiAlCl_4_ occupy tetra­hedral inter­stices with site symmetries *m* and 1, respectively, the lithium cation site being half-occupied (Figs. 1 ▸ *b* and 1*c*). Hence, the solid state of the title compound represents a three-dimensional network of corner-sharing tetra­hedra, while in LiAlCl_4_(*mP*24), the octa­hedral and tetra­hedral polyhedra are connected *via* corners as well as edges. LiAlCl_4_(*oP*12) exhibits, as expected, shorter Li—Cl bonds (coordination number 4) as compared to corresponding bonds in monoclinic LiAlCl_4_ (coordination number 6). Using the Brown formalism (Brown & Altermatt, 1985 ▸), in both cases, bond orders which differ significantly from the expected value in view of the monovalent cation are computed (Table 1 ▸). In the case of ortho­rhom­bic LiAlCl_4_, the strong deviation is based on the statistical disorder mentioned above and corresponding averaged geometric parameters obtained for occupied and non-occupied tetra­hedral inter­stices, leading to higher Li—Cl bond orders in view of the exponential relationship between bond length and bond order.

## Raman spectra   

**Figure 2:**
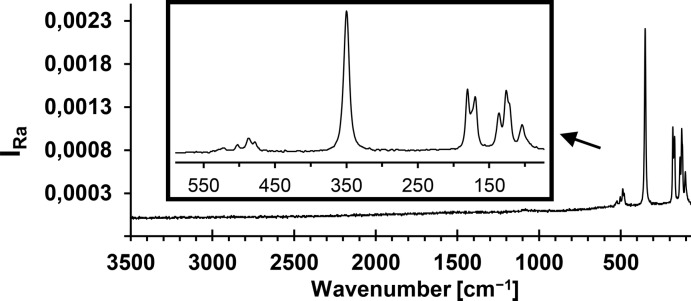
Raman spectrum of the title compound.

Raman bands in the vibrational spectrum of the title compound (Fig. 2 ▸) can be assigned to the four normal modes of vibration of a five atomic tetra­hedral moiety of composition *AX*_4_ (Nakamoto, 1986 ▸) ν_s_(*A*_1_: 350 cm^−1^), δ_d_(*E*: 136 and 126 cm^−1^), ν_d_(*F*_2_: 523, 502, 487 and 478 cm^−1^) and δ_d_(*F*_2_: 180 and 170 cm^−1^). As in the Raman spectra of other alkali metal tetra­chlorido­aluminates (Rytter & Øye, 1973 ▸; Rubbens *et al.*, 1978 ▸) or NH_4_AlCl_4_ (Mairesse *et al.*, 1978*b* ▸), splitting of the bands is observed corresponding to the site effect and perturbation of the ideal tetra­hedral symmetry of free AlCl_4_^−^ anions caused by cation inter­actions.

## Thermal analysis and X-ray powder diffraction   

**Figure 3:**
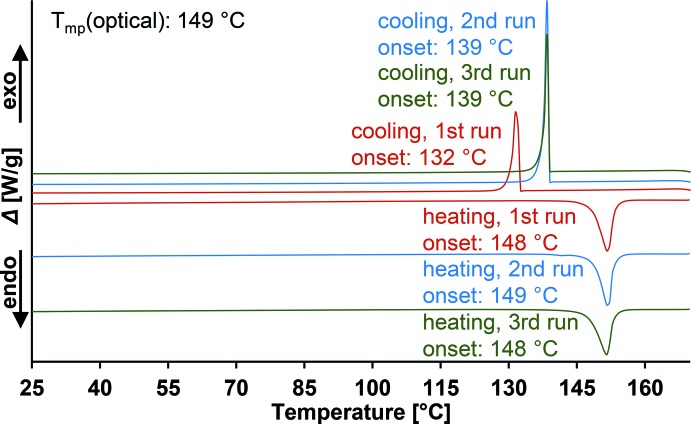
DSC curves of multiple runs of the title compound.

**Figure 4:**
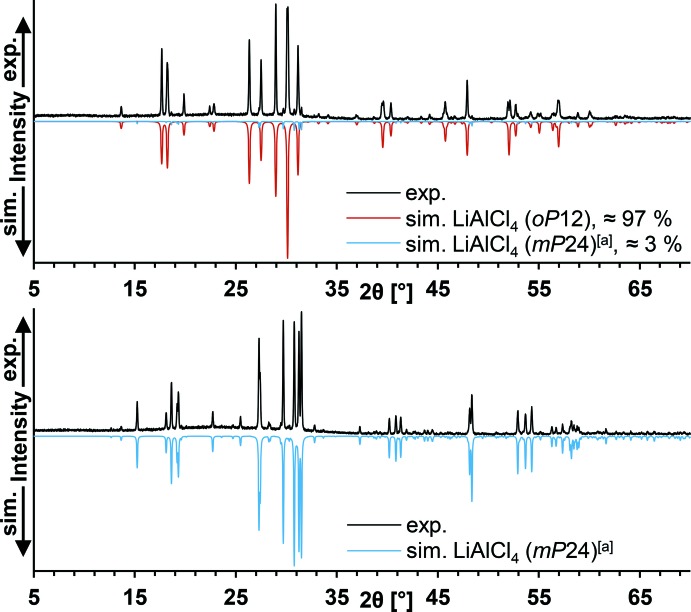
X-ray powder diffraction pattern of the title compound before (top) and after (bottom) melting and corresponding simulations. [*a*] Single-crystal data for LiAlCl_4_(*mP*24) are taken from the literature (Perenthaler *et al.*, 1982 ▸).

From DSC measurements of the title compound (Fig. 3 ▸), no evidence for a phase transition is found until the material melts at 148 °C (*T*_peak_ = 152 °C). The melting point is nearly identical to literature data for LiAlCl_4_(*mP*24) (146 °C; Weppner & Huggins, 1976 ▸), which seems to be the only modification that recrystallizes from the melts of both modifications. This is demonstrated by high-quality X-ray powder diffraction patterns of the title compound, crystallized from *para*-xylene solution, and of the crystalline solid obtained by recrystallization from the melt (Fig. 4 ▸). In view of the current data, we suppose LiAlCl_4_(*oP*12) to represent a metastable phase of lithium tetra­chlorido­aluminate whose melting point probably is nearly identical to that of monoclinic LiAlCl_4_ because it is very unlikely that a phase transition would not have been observed with the chosen DSC methods. The lower density of ortho­rhom­bic LiAlCl_4_ (1.89 g cm^−3^) compared to monoclinic LiAlCl_4_ (1.98 g cm^−3^; Mairesse *et al.*, 1979 ▸) supports the assumption of its metastability.

## Synthesis and crystallization   

All sample preparations and manipulations were carried out in an atmosphere of dry argon (argon 5.0) using either Schlenk techniques or an MBraun LABstar glove-box. LiCl (beads, 99.9+%, anhydrous) and AlCl_3_ (powder, 99.99%) were purchased from Sigma–Aldrich and while LiCl was used as received, AlCl_3_ was first overlayed with elemental aluminium (grit, ≥97%, Sigma–Aldrich) and sublimed in a sealed ampoule in *vacuo* at 190 °C. *p*-Xylene (99%, Sigma–Aldrich) and *m*-xylene (99%, TCI) were refluxed with aluminium chloride, washed with 0.2 *M* NaOH, as well as distilled water, and distilled on mol­ecular sieve 4 Å afterwards. In a typical reaction, 0.112 g (2.64 mmol) lithium chloride and 0.268 g (2.01 mmol) aluminium chloride were treated with 5 ml *p*-xylene and the mixture was refluxed for 30 min. Seperation of the warm colourless solution from residual LiCl and removal of 4 ml of the solvent under reduced pressure at room temperature led to the formation of colourless crystals of the title compound. LiAlCl_4_(*oP*12, *Pmn*2_1_) was isolated in 60% yield after washing the crystalline material with *p*-xylene and drying the solid in *vacuo* at room temperature.

The FT–Raman spectrum was recorded using a Bruker MultiRam spectrometer (*OPUS*; Bruker, 2006 ▸) equipped with an RT-InGaAs-detector and an Nd:YAG-Laser at 1064 nm (Stokes: 3500–70 cm^−1^; resolution: 2 cm^−1^): ν_d_(*F*_2_, AlCl_4_^−^): 523 (*w*), 502 (*w*), 487 (*m*), 478 (*w*); ν_s_(*A*_1_, AlCl_4_^−^): 350 (*vs*); δ_d_(*F*_2_, AlCl_4_^−^): 180 (*s*), 170 (*s*); δ_d_(*E*, AlCl_4_^−^): 136 (*m*), 126 (*s*), 104 (*m*).

Thermal analysis (differential scanning calorimetry) was carried out with a Mettler Toledo DSC 1 calorimeter (*STARe*; Mettler-Toledo, 2008 ▸) equipped with an FRS 5 sensor using medium pressure steel crucibles without sealing rings. Measurements were carried out in an atmosphere of dry nitro­gen at a heating/cooling rate of 5 °C min^−1^ between 0 and 170 °C. First measurement heating: *T*_onset_ = 148 °C (*T*_peak_ = 152 °C), endothermic, melting; first measurement cooling: *T*_onset_ = 132 °C (*T*_peak_ = 132 °C), exothermic, crystallization; second measurement heating: *T*_onset_ = 149 °C (*T*_peak_ = 152 °C), endothermic, melting; second measurement cooling: *T*_onset_ = 139 °C (*T*_peak_ = 138 °C), exothermic, crystallization; third measurement heating: *T*_onset_ = 148 °C (*T*_peak_ = 152 °C), endothermic, melting; third measurement cooling: *T*_onset_ = 139 °C (*T*_peak_ = 139 °C), exothermic, crystallization. An alternative melting-point determination was carried out with a Mettler Toledo MP 90 Melting Point System: *T*_mp_ = 149 °C.

X-ray powder diffraction patterns were measured using a Stoe & Cie STADI P (*WinXPOW*; Stoe & Cie, 2003 ▸) Debye–Scherrer diffractometer working in transmission mode with Cu *Kα*_1_ radiation [Ge(111) monochromator]. Simulations of powder patterns from single-crystal data were carried out using the computer program *PowderCell* (Kraus & Nolze, 2000 ▸).

## Refinement   

Crystal data, data collection and structure refinement details are summarized in Table 2 ▸. The lithium cation site (general position, Wyckoff site 4*b*) is half occupied.

## Supplementary Material

Crystal structure: contains datablock(s) I. DOI: 10.1107/S205698901701235X/wm5410sup1.cif

Structure factors: contains datablock(s) I. DOI: 10.1107/S205698901701235X/wm5410Isup2.hkl

CCDC reference: 1570829

Additional supporting information:  crystallographic information; 3D view; checkCIF report

## Figures and Tables

**Table 1:** Selected bond lengths (Å) in LiAlCl_4_(*oP*12) and LiAlCl_4_(*mP*24) (Perenthaler *et al.*, 1982 ▸) in the left and right column, respectively, and corresponding sums of bond orders, calculated using the Brown formalism (*r*_0_ = 1.91, B = 0.37; Brese & O’Keeffe, 1991 ▸)

Li1—Cl1	2.322 (17)	Li—Cl1	2.475 (7)
Li1—Cl2	2.381 (21)	Li1—Cl2^*x*^	2.729 (7)
Li1—Cl2^i^	2.356 (14)	Li1—Cl2^xi^	2.841 (7)
Li1—Cl3	2.413 (17)	Li1—Cl3	2.594 (7)
		Li1—Cl3^xii^	2.769 (7)
		Li1—Cl4^xiii^	2.493 (7)
Σs(Li—Cl)	1.17	Σs(Li—Cl)	0.87

Symmetry codes: (i) −*x* + 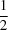, −*y*, *z* − 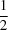; (*x*) −*x*, *y* − 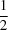, −*z* + 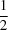; (xi) *x*, −*y* + 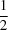, *z* + 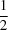; (xii) −*x*, *y* − 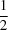, −*z* + 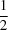; (xiii) 1 − *x*, *y* − 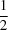, −*z* + 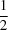.

**Table 2:** Experimental details

Crystal data	
Chemical formula	LiAlCl_4_
*M*_r_	175.72
Crystal system, space group	Orthorhombic, *P* *m* *n*2_1_
Temperature (K)	173
*a*, *b*, *c* (Å)	7.8273 (10), 6.4466 (10), 6.1304 (8)
*V* (Å^3^)	309.34 (7)
*Z*	2
Radiation type	Mo *K*α
μ (mm^−1^)	1.90
Crystal size (mm)	0.65 × 0.10 × 0.03

Data collection	
Diffractometer	Stoe IPDS 2T
Absorption correction	Multi-scan (*X-AREA*; Stoe & Cie, 2009 ▸)
*T*_min_, *T*_max_	0.431, 0.583
No. of measured, independent and observed [*I* > 2σ(*I*)] reflections	3388, 880, 870
*R*_int_	0.092
(sin θ/λ)_max_ (Å^−1^)	0.685

Refinement	
*R*[*F* ^2^ > 2σ(*F* ^2^)], *wR*(*F* ^2^), *S*	0.028, 0.075, 1.21
No. of reflections	880
No. of parameters	37
No. of restraints	1
Δρ_max_, Δρ_min_ (e Å^−3^)	0.48, −0.46
Absolute structure	Flack *x* determined using 386 quotients [(*I* ^+^) − (*I* ^−^)]/[(*I* ^+^) + (*I* ^−^)] (Parsons *et al.*, 2013 ▸)
Absolute structure parameter	0.1 (2)

Computer programs: *X-AREA* (Stoe & Cie, 2009 ▸), *SHELXT* (Sheldrick, 2015*a* ▸), *SHELXL2014* (Sheldrick, 2015*b* ▸), *DIAMOND* (Brandenburg, 2016 ▸) and *publCIF* (Westrip, 2010 ▸).

## supplementary crystallographic information

### Crystal data

**Table:**

LiAlCl_4_	*D*_x_ = 1.887 Mg m^−^^3^
*M**_r_* = 175.72	Mo *K*α radiation, λ = 0.71073 Å
Orthorhombic, *P**m**n*2_1_	Cell parameters from 6814 reflections
*a* = 7.8273 (10) Å	θ = 3.0–29.7°
*b* = 6.4466 (10) Å	µ = 1.90 mm^−^^1^
*c* = 6.1304 (8) Å	*T* = 173 K
*V* = 309.34 (7) Å^3^	Needle-shaped, colorless
*Z* = 2	0.65 × 0.10 × 0.03 mm
*F*(000) = 168	

### Data collection

**Table:**

Stoe IPDS 2T diffractometer	870 reflections with *I* > 2σ(*I*)
Radiation source: fine-focus sealed tube	*R*_int_ = 0.092
φ–scans	θ_max_ = 29.1°, θ_min_ = 3.2°
Absorption correction: multi-scan (X-AREA; Stoe & Cie, 2009)	*h* = −10→10
*T*_min_ = 0.431, *T*_max_ = 0.583	*k* = −8→8
3388 measured reflections	*l* = −8→8
880 independent reflections	

### Refinement

**Table:**

Refinement on *F*^2^	1 restraint
Least-squares matrix: full	*w* = 1/[σ^2^(*F*_o_^2^) + (0.022*P*)^2^ + 0.166*P*] where *P* = (*F*_o_^2^ + 2*F*_c_^2^)/3
*R*[*F*^2^ > 2σ(*F*^2^)] = 0.028	(Δ/σ)_max_ < 0.001
*wR*(*F*^2^) = 0.075	Δρ_max_ = 0.48 e Å^−^^3^
*S* = 1.21	Δρ_min_ = −0.46 e Å^−^^3^
880 reflections	Absolute structure: Flack *x* determined using 386 quotients [(I+)-(I-)]/[(I+)+(I-)] (Parsons *et al.*, 2013)
37 parameters	Absolute structure parameter: 0.1 (2)

### Special details

**Table:**

Geometry. All esds (except the esd in the dihedral angle between two l.s. planes) are estimated using the full covariance matrix. The cell esds are taken into account individually in the estimation of esds in distances, angles and torsion angles; correlations between esds in cell parameters are only used when they are defined by crystal symmetry. An approximate (isotropic) treatment of cell esds is used for estimating esds involving l.s. planes.

### Fractional atomic coordinates and isotropic or equivalent isotropic displacement parameters (Å^2^)

**Table:**

	*x*	*y*	*z*	*U*_iso_*/*U*_eq_	Occ. (<1)
Li1	0.246 (2)	0.1671 (19)	0.493 (3)	0.035 (3)	0.5
Cl1	0.0000	0.31452 (19)	0.34835 (19)	0.0305 (3)	
Cl2	0.22483 (12)	0.18042 (11)	0.88033 (12)	0.0317 (2)	
Cl3	0.5000	0.35454 (16)	0.3896 (3)	0.0295 (3)	
Al1	0.0000	0.3312 (2)	−0.0004 (3)	0.0229 (3)	

### Atomic displacement parameters (Å^2^)

**Table:**

	*U*^11^	*U*^22^	*U*^33^	*U*^12^	*U*^13^	*U*^23^
Li1	0.043 (8)	0.033 (6)	0.029 (8)	0.001 (6)	0.002 (6)	0.000 (4)
Cl1	0.0300 (5)	0.0405 (6)	0.0208 (7)	0.000	0.000	0.0010 (4)
Cl2	0.0313 (4)	0.0314 (4)	0.0324 (5)	0.0078 (3)	0.0067 (5)	0.0017 (5)
Cl3	0.0300 (5)	0.0226 (4)	0.0360 (6)	0.000	0.000	−0.0055 (6)
Al1	0.0244 (8)	0.0212 (6)	0.0233 (8)	0.000	0.000	0.0008 (5)

### Geometric parameters (Å, º)

**Table:**

Li1—Cl1	2.322 (17)	Cl2—Li1^iv^	2.356 (14)
Li1—Cl2^i^	2.356 (14)	Cl3—Al1^v^	2.1354 (18)
Li1—Cl2	2.38 (2)	Cl3—Li1^vi^	2.413 (17)
Li1—Cl3	2.413 (17)	Al1—Cl3^vii^	2.1354 (18)
Cl1—Al1	2.1404 (19)	Al1—Cl2^viii^	2.1392 (11)
Cl1—Li1^ii^	2.322 (17)	Al1—Cl2^ix^	2.1392 (11)
Cl2—Al1^iii^	2.1392 (11)		
			
Cl1—Li1—Cl2^i^	111.0 (7)	Li1^iv^—Cl2—Li1	104.6 (5)
Cl1—Li1—Cl2	108.0 (7)	Al1^v^—Cl3—Li1^vi^	113.1 (4)
Cl2^i^—Li1—Cl2	109.5 (6)	Al1^v^—Cl3—Li1	113.1 (4)
Cl1—Li1—Cl3	112.2 (6)	Li1^vi^—Cl3—Li1	111.1 (7)
Cl2^i^—Li1—Cl3	108.6 (7)	Cl3^vii^—Al1—Cl2^viii^	108.85 (6)
Cl2—Li1—Cl3	107.5 (7)	Cl3^vii^—Al1—Cl2^ix^	108.85 (6)
Al1—Cl1—Li1^ii^	113.7 (5)	Cl2^viii^—Al1—Cl2^ix^	110.70 (8)
Al1—Cl1—Li1	113.7 (5)	Cl3^vii^—Al1—Cl1	111.28 (9)
Li1^ii^—Cl1—Li1	111.9 (9)	Cl2^viii^—Al1—Cl1	108.58 (6)
Al1^iii^—Cl2—Li1^iv^	114.3 (4)	Cl2^ix^—Al1—Cl1	108.58 (6)
Al1^iii^—Cl2—Li1	114.4 (4)		

Symmetry codes: (i) −*x*+1/2, −*y*, *z*−1/2; (ii) −*x*, *y*, *z*; (iii) *x*, *y*, *z*+1; (iv) −*x*+1/2, −*y*, *z*+1/2; (v) −*x*+1/2, −*y*+1, *z*+1/2; (vi) −*x*+1, *y*, *z*; (vii) −*x*+1/2, −*y*+1, *z*−1/2; (viii) −*x*, *y*, *z*−1; (ix) *x*, *y*, *z*−1.
